# Who Gains Mental Health Benefits from Work Autonomy? The Roles of Gender and Occupational Class

**DOI:** 10.1007/s11482-023-10161-4

**Published:** 2023-03-07

**Authors:** Zhuofei Lu, Senhu Wang, Yaojun Li, Xiyuan Liu, Wendy Olsen

**Affiliations:** 1 Department of Social Statistics, University of Manchester, HBS Building, Oxford Road, Manchester, M13 9PL UK; 2 Department of Sociology, National University of Singapore, 11 Arts Link, #03-06 AS1, Singapore, 117573 Singapore; 3 Department of Sociology and Cathie Marsh Institute for Social Research, University of Manchester, HBS Building, Oxford Road, Manchester, M13 9PL UK; 4 Department of Sociology, University of Manchester, Arthur Lewis Building, Oxford Road, Manchester, M13 9PL UK; 5 Department of Social Statistics and Cathie Marsh Institute for Social Research, University of Manchester, HBS Building, Oxford Road, Manchester, M13 9PL UK

**Keywords:** Job quality, Mental health, Occupational statuses, Work autonomy, Workplace interventions

## Abstract

In recent years, improving work autonomy as an important priority in the UK labour market has been shown to enhance employee mental health and well-being. However, previous theories and empirical studies have paid little attention to the intersectional inequalities in the mental health benefits of work autonomy, preventing us from gaining a comprehensive understanding of the mental consequences of work autonomy. By integrating literature from occupational psychology, gender and social class, this study develops theoretical hypotheses regarding whether and how the mental health benefits of work autonomy vary alongside the intersectional axes of gender and occupational class and tests these hypotheses using long-term panel data in the UK (2010–2021). Overall, we find that those from higher occupational class and male employees acquire significantly more mental health benefits from high work autonomy compared with those from lower occupational class and female employees. Moreover, further analyses show significant intersectional inequalities of gender and occupational class. While male employees from all occupational classes gain significant mental health benefits from work autonomy, only female employees from higher (but not lower) occupational classes benefit from work autonomy. These findings contribute to the literature in the sociology of work by demonstrating the intersectional inequalities in mental health consequences of work autonomy, especially for women in the lower occupational class, highlighting the need for a more gender- and occupation-sensitive design in future labour market policies.

## Introduction

Work autonomy is defined as the control that workers have over decisions within their jobs, such as the control over work pace, task orders and schedule (Fielding, 1990; Wheatley, 2017). According to the summary from the ‘Work Autonomy, Flexibility and Work-Life Balance’ project funded by the Economic and Social Research Council, some common flexible working arrangements (i.e., flexible schedules and teleworking) provide workers with more working time autonomy and also enhance their control over their work pace and task orders (Chung, 2017). This implies a trend of the conflation of work autonomy with formal flexible working arrangements. In recent decades, work autonomy has not only been found important for employees’ job performance and work commitments, but has also been found to improve employees’ mental health (Chung, 2017; Lopes et al., 2014; Wheatley, 2017; Yunus & Mostafa, 2021). Thus, improving work autonomy has become an important objective of current occupational health and welfare policies.

Over the last decade, labour market policies in the UK have significantly increased access to flexible working and the autonomy of work, providing a new context to the research on work autonomy. Before 2014, British employees who requested flexible working were disproportionately from certain demographic groups, such as parents with childcare responsibilities (Chung & van der Horst, 2020), which was a very small proportion. Since 2014, the UK government has extended the right to request flexible work to all employees in the UK. Although there is a bit of difference between work autonomy and flexible working practices, the expansion of flexible working inevitably promotes work autonomy in the UK labour market. As a result, it is no longer certain whether work autonomy will benefit the general working population due to the increased diversity of national workers who have work flexibility and autonomy. For example, some research suggests that there are some negative consequences of work autonomy (e.g. blurred work-family boundary and flexibility stigma), which may offset or even outweigh its mental health benefits (Chung, 2018; Williams et al., 2013). Thus, the first objective of this research is to re-investigate the relationship between paid work autonomy and mental well-being across the general working population using updated panel data (2010–2021) from the UK.

Moreover, it is expected that how work autonomy shapes mental health depends on gender and social class due to widely argued gender and class differences in labour force participation and cultural norms. Regarding gender differences, the doing gender theory argues that due to persistent traditional gender norms, work autonomy may intensify gender inequality by increasing women’s unpaid work hours and work-family conflicts (Chung & van der Horst, 2020; West & Zimmerman, 1987). In addition, scholars argue that female workers with more work flexibility or autonomy may actually work longer and have more work-family conflicts, which is termed as the ‘flexibility paradox’ (Chung, 2022; Glavin & Schieman, 2012; Mazmanian et al., 2013). For instance, Chung and her colleagues found that women tend to have more family-work or work-family conflicts when working from home, not for men (Yucel & Chung, 2021). Therefore, the mental benefits of work autonomy might be more pronounced among male employees. Regarding class differences, studies indicate that different occupational groups have varying levels of work demands and task complexity, and that higher levels of work demands and complexity lead to more mental stress and role conflicts (Glavin & Schieman, 2012; Schieman et al., 2006, 2009). Thus, the mental health effects of work autonomy may be more pronounced among the higher occupational classes, that is, employees in managerial or professional roles. Furthermore, there is a potential intersection of gender and occupational class where men and women in different occupational classes have different identities and work and family demands. Therefore, the second objective of this article is to examine whether and to what extent the influence of work autonomy differs across different gender and occupational classes.

By achieving both objectives, this study contributes to the literature in two important ways. Firstly, the study uses fixed-effects models to analyse the samples of general British employees over a long period of time, improving the generalizability of previous research. Secondly, by analysing the variations between different gender and occupational classes, this article facilitates a more nuanced understanding of the intersection of gender and social class in the associations between work autonomy and mental well-being, which has significant implications for future policy interventions. Specifically, the study’s findings indicate that female employees in the lower occupational classes are most disadvantaged in the mental consequence of work autonomy, which is a pressing matter that needs to be addressed properly in the current labour market. The policymakers should not only focus on the overall effect of improving work autonomy but also pay attention to the intensified mental health inequalities during the process.

### Work Autonomy and Mental Well-Being

Previous research from occupational psychology shows that work autonomy can improve workers’ mental health and well-being by promoting work-life balance and job efficiency (Chung, 2017; Lott & Chung, 2016; Wheatley, 2017). Two core theories that have been widely used to interpret the mental benefits of work autonomy are the Job-Demand Control (JDC) model (Karasek, 1979) and the theory of work-family conflict (Aryee, 1992; Chandola et al., 2019).

The JDC model indicates that high levels of work autonomy can improve employees’ job quality and decrease stress levels. Specifically, the JDC model identifies four types of jobs based on relative job control and demand. There are ‘active jobs’ with high autonomy and high demand; ‘high-strain jobs’ with low work autonomy and high demand; ‘passive jobs’ with low work autonomy and low demand; and finally ‘low-strain jobs’ with high work autonomy and low demand (Karasek, 1979). According to the arguments based on the JDC model, high work autonomy in ‘active jobs’ can effectively alleviate the stress employees feel due to high work demand (Karasek, 1979). For instance, previous studies have found that higher levels of work autonomy can improve employees’ job satisfaction while relieving work-related stress (Chung, 2017; Clausen et al., 2021; Grönlund, 2007; Kalleberg et al., 2009). Therefore, we expect that work autonomy can improve employees’ mental well-being by ensuring good job quality and alleviating the mental strain brought by work demands.

Similarly, arguments based on the work-family conflict theory indicate that work autonomy can alleviate the conflicts between employees’ work and family life, thereby benefiting employees’ mental health and well-being (Chandola et al., 2019; Glavin & Schieman, 2012; Jeffrey Hill et al., 2008a). According to role strain theory (Goode, 1960), individuals’ multiple social roles compete for finite resources (i.e., time and energy) and are frequently conflicting with each other (Aryee, 1992). Such forms of inter-role conflict are associated with higher levels of stress and increased psychological strain (Chandola et al., 2019; Wang et al., 2022c). For instance, a strand of research shows that work-family conflict can lead to a series of mental health problems, such as burnout, depression, anxiety and mental distress (Enns et al., 2015; Jeffrey Hill et al., 2008b; Takahashi et al., 2011). Extensive studies from many different countries (including Sweden, the UK and the US) indicate that work autonomy enables employees to balance conflicting expectations and demands from the work and family spheres (Chung, 2017; Chung & van der Lippe, 2018; Glavin & Schieman, 2012; Grönlund, 2007). Therefore, organisations around the world have identified the alleviation of work-family conflicts as an effective way to promote employees’ mental well-being (L. Z. Li & Wang, 2022). Taken together, we expect that work autonomy will improve employees’ mental well-being by alleviating the conflicts between work and life.

- Hypothesis 1: Employees with more work autonomy have better mental well-being than their counterparts.

### Gender Disparities

Given the widely acknowledged gender differences in labour market experience, it is important to explore whether the effects of work autonomy on mental well-being vary between genders. When examining the mental consequences of different workplaces, previous research usually considers potential gender disparities and analyses the samples by gender (Chandola et al., 2019; Wang et al., 2022b). In our case, we expect both male and female employees to benefit from work autonomy, but the benefits might be more pronounced among male employees.

Both men and women can benefit from work autonomy but in different mechanisms. As for women, work autonomy enables them to balance their work and family commitments. Due to the traditional gender norms (Risman, 2004) and family devotion schema (Blair-Loy 2009) in the UK, women are expected to take on more domestic responsibilities and child-rearing and are very likely to face more work-family conflicts than men (Kan, 2007; Kan & Laurie, 2018; Yucel & Chung, 2021). Thus, work autonomy is promoted to alleviate female employees’ work-family conflicts and reduce their work-related stress and time pressure (Craig & Brown, 2017; Wheatley, 2017). As for men, work autonomy can help them alleviate the mental strain brought by the high demands of their job. Given that male employees are more satisfied with ‘active jobs’ (Karasek, 1979), which are highly demanding, male employees may face more job-related stress. This can be seen in the differences in the career expectations between men and women. Previous research on the career expectations of employees found that female employees prioritise their work-life balance, while males prioritise ‘high salary’ prospects (Schweitzer et al., 2011). Overall, both men and women suffer a series of mental issues (i.e., feelings of stress, subjective time pressure and anxiety) in both the work and family spheres but through different mechanisms. Drawn on the predictions from the JDC model and the role strain theory, work autonomy might alleviate these mental issues to promote mental well-being.

However, there are a series of potential adverse effects accompanied by the benefits of work autonomy, especially among women. A strand of studies found that females with more work autonomy tend to suffer low time quality and enjoyment at work, especially for female teleworkers (Craig & Brown, 2017; Lu & Zhuang, 2023; Powell & Craig, 2015). This is because women are more likely to use work autonomy to facilitate family demands (Abendroth, 2022; Kim et al., 2019), which can increase their frequency of multitasking. For instance, a mother with more control over work schedules and pace can choose to settle family matters during the normal working period (i.e., 3 pm–5 pm), while this can make their work schedules more fragmented and increase subjective time pressure (Cornwell, 2013; Craig & Brown, 2017). In addition, homeworkers with the presence of children tend to have more multitasking time episodes to address the conflicts between work and childcare responsibilities. Multitasking and the spillover effects of work to the private sphere can lead to higher stress, burnout and anxiety levels. On the one hand, women (especially mothers) can benefit from work autonomy in addressing dilemmas between work and family. On the other hand, since women with more work autonomy are more likely to have their work time disrupted in ways that are unexpected and ‘family-oriented’, the potential adverse mental effects can partially offset the benefits of work autonomy. As for men, they are more likely to use work autonomy to benefit themselves outside of the family context, which gives them a lower risk of momentarily increased workloads and work-family conflict. In sum, we expect that the mental benefits of work autonomy are more pronounced among male employees than female employees.

- Hypothesis 2: The mental benefits of work autonomy on mental health are more pronounced among male employees.

### Occupational Class Disparities

Employees in different occupational classes have varying levels of job demands and complexity, which can result in different levels of inter-role conflict and mental strain. Specifically, Goldthorpe’s class theory indicates that social class has distinct explanatory power when it comes to studying the consequence of work conditions (Breen & Rottman, 1995; Chan & Goldthorpe, 2007). For example, workers in the professional and managerial classes have service contracts associated with long-term rewards and more autonomy, while the lower occupational groups have labour contracts associated with less job security, autonomy and prospects (Savage et al., 2013). Thus, the mental health benefits of work autonomy may vary across different occupational classes. We expect that the benefits of work autonomy on employees’ mental well-being will be more pronounced among the higher occupational classes, especially for the professional and managerial classes, for the following reasons.

The first reason is that though the professional and managerial occupational class correlates with greater work autonomy, it also brings higher demands and expectations, leading to more inter-role conflicts and stress. Employees in the higher occupational classes have more inter-role conflicts and are more likely to be constrained by norms of how ideal workers should behave (Schieman et al., 2009). Specifically, the ideal worker norm expects workers to prioritise their work over family responsibilities by working long hours and having a strong work ethic. Thus, employers are more likely to promote the ideal worker norm among employees in the higher occupational classes (Schieman et al., 2006). Accordingly, employees in the higher occupational classes need more work autonomy to balance their work and home life. There is ample research to show that employees in the higher occupational classes (particularly the professional and managerial groups) have more work-family conflicts than those in lower occupational classes (Glavin & Schieman, 2012; Schieman et al., 2006, 2009). Therefore, we expect that work autonomy might have a more pronounced benefit for the mental well-being of professional and managerial workers.

The second reason we expect work autonomy to have a greater benefit for workers in higher occupation classes is that high-ranking jobs in institutions often involve more complex tasks. Thus, workers in these roles need more work autonomy to increase their job performance and self-encouragement, thereby improving their mental well-being. Previous studies have found that the higher demand for work autonomy amongst workers in the professional and managerial occupational class is due to the nature of the work itself (Mastekaasa, 2011; Weeden & Grusky, 2005). Employees with high occupational statuses, such as professors, business managers, and lawyers can have a higher risk of depression or anxiety without high work autonomy, because their work itself needs more creativity and authority. Therefore, work autonomy might improve the job quality of employees in the professional and managerial occupational class, thereby benefiting their job-related well-being. Taken together, we argue that the mental benefits of work autonomy are more pronounced among employees in the professional and managerial occupational class, and so formulate the following hypothesis:

- Hypothesis 3: Employees in the higher occupational classes gain more mental-health benefits from work autonomy than their counterparts.

Furthermore, in line with the discussion in “Gender Disparities” section, we consider the potential intersection of gender and class and expect that females in the lower occupational classes benefit the least from work autonomy, thereby suffering double jeopardy. Specifically, in the lower occupational classes, female employees need work autonomy to improve their work-family balance, while they always suffer the worst work conditions and disadvantaged positions in the household. First, the disadvantaged positions of females in the lower occupational classes place a “glass ceiling” on their opportunities for promotion into higher positions that offer more work autonomy (Clawson, 2014; England et al., 2020). Second, studies indicate that women in the lower occupational classes tend to take on more housework than the higher occupational groups since they have less bargaining power in both the labour market and households (Dumont et al., 2012; Kan & He, 2018). For instance, studies on the gender and class disparities in the division of labour and work flexibility/autonomy indicate that women in lower occupational classes are more likely to sacrifice the benefits of work autonomy to maximise family time/childcare (Chung & Booker, 2022; Kim, 2020). Third, a strand of studies finds that women in lower occupational classes tend to have more traditional gender role attitudes, which are in direct conflict with the ideal worker norms that workers should prioritise their work commitments (Cha & Weeden, 2014). By contrast, though women in higher occupational classes may still be constrained by gender norms, work autonomy can promote their labour participation by preventing them from falling into the more traditional divisions of labour (Chung & Booker, 2022; Wang & Lu, 2022). Therefore, the potential adverse effects of work autonomy can be more pronounced among females in the lower occupational classes since they are more likely to sacrifice the benefits brought by work autonomy to facilitate family demands.

As for men, especially those in the higher occupational groups, work autonomy may adversely affect male employees’ mental health by increasing their work intensity or working hours, which is termed ‘the autonomy paradox’ (Chung & van der Horst, 2020; Mazmanian et al., 2013). This is because men in the higher occupational classes are more likely to be restricted by the work devotion schema, thereby being willing to work more in exchange for employers’ rewards (work autonomy) (Bathini & Kandathil, 2019). However, a stream of the latest research indicates that it might be job quality rather than job quantity that is important for workers’ mental health (Wang et al. 2022a; Wang & Li, 2022). Specifically, job quality and the discrepancy between actual and preferred working hours (termed as working time mismatches) can be more important in predicting workers’ mental health status than actual working hours (Golden & Gebreselassie, 2007; Kamerāde et al., 2019). Indeed, previous studies suggest that men in the higher occupational classes have significantly longer working hours and job demands than those in the lower occupations (Hoven et al., 2015; Kawakami et al., 2004). Work autonomy as a vital index of job quality might buffer the potential adverse effects of longer working hours on mental health. As a result, work autonomy can be more vital for male employees in the higher occupational classes as it enables them to alleviate their higher workloads/job demands and intensity, thereby benefiting their mental health. Taken together, we expected that the mental benefits of work autonomy could be least pronounced among lower occupational females but most pronounced among professional & managerial males.

- Hypothesis 4: Female employees in the higher occupational classes gain more mental-health benefits from work autonomy than the lower occupational groups.
- Hypothesis 5: Male employees in the higher occupational classes gain more mental-health benefits from work autonomy than the lower occupational groups.

## Method

### Data and Sample

This study used the second (2010–2011), fourth (2012–2013), sixth (2014–2015), eighth (2016–2017), tenth (2018–2019) and twelfth (2020–2021) waves of the United Kingdom Household Longitudinal Study (UKHLS), which is a widely used source of household panel data in the UK (University of Essex, 2022). The UKHLS includes a sample of around 40,000 households in the first wave, which was collected using a stratified and clustered sampling method. The UKHLS began collecting respondents’ information about work autonomy in 2010, while the latest wave that includes information about work autonomy is the twelfth (2020–2021). Only these six waves of the UKHLS cover all the key variables we need. The average interview response rate (adult) was around 60% among the five waves. In line with previous research on work autonomy and flexible working (Chung & van der Horst, 2020; Li & Wang, 2022; Wheatley, 2017), the samples used in this study excluded respondents aged under 18 or over 65, those who were not in paid employment, and those who were self-employed. We also excluded all the missing cases (around 15%). The final analytic sample includes 93,903 observations. Table 1 reports the sample descriptive statistics of the baseline wave (2010–2011).

**Table 1:** Sample descriptive statistics at the baseline wave (2010–2011)

Variables	%, Mean	SD	Min	Max
Mental distress (reversed GHQ-12)	25.3	4.8	0	36
Work autonomy	3.0	0.8	1	4
Age	41.4	11.7	18	65
Sex				
Male	45.0%			
Female	55.0%			
The occupational class, %				
High	44.8%			
Middle	25.8%			
Low	29.4%			
Marital status				
Never married/Single	18.6%			
Married	72.1%			
Divorced/separated/widowed	9.3%			
The presence of dependent children				
No	59.2%			
Yes	40.8%			
The presence of long-standing illness				
Yes	23.8%			
No	76.2%			
Number of children in the household	0.7	0.9	0	7
Log personal income (monthly)	7.6	0.5	–1.2	10.4
Job satisfaction	5.3	1.4	1	7
Job-related anxiety	1.8	0.8	1	5

% = Proportion, M = Mean, SD = Standard deviation; the number of observations in the baseline wave is 19,653 (unbalanced panel)

### Measurements

#### Outcome Variables

*Mental health* is measured by the widely used (Li & Wang, 2022; Li, 2016) and validated 12-item General Health Questionnaire (GHQ-12). The questions included in the GHQ-12 measure respondents’ general mental distress, such as symptoms of depression and anxiety, sleeping problems, and overall happiness (Goldberg & Hillier, 1979). In the UKHLS, respondents’ answers to the GHQ-12 were converted to a single continuous scale ranging from 0 (the least distressed) to 36 (the most distressed), with a higher score indicating worse mental health (more distress). The Cronbach’s Alpha is 0.89, suggesting that the GHQ-12 items have a high level of internal consistency. We reverse the scale to conveniently interpret the results, with a higher score indicating better mental health.

#### Independent Variables

*Work autonomy* is measured by the total score of five different work autonomy variables: (1) autonomy over job tasks; (2) autonomy over job pace; (3) autonomy over job hours; (4) autonomy over job order; and (5) autonomy over job manner. These five autonomy variables are measured on a scale ranging from 1 (a lot) to 4 (none). For this measurement, Cronbach’s Alpha is 0.84, suggesting that the work autonomy items have a high level of internal consistency. In line with previous research (Chandola & Zhang, 2018), work autonomy is summed and adjusted to a continuous variable ranging from 1 (none) to 4 (a lot). Although work autonomy and flexible working have many similarities in practice (i.e., flextime and schedule control) (Chung & van der Horst, 2020), there are still differences between the two types. Specifically, flexible working practices generally reflect work-family intervention policies in the workplace, while work autonomy practices are identified as ‘employer-friendly’ policies that aim to improve workers’ job efficiency instead of work-family balance (Chung & van der Lippe, 2018). In addition, different from most studies on flexible working that use access and the use of flexible working as the predictor of mental well-being (Chandola et al., 2019; Wang & Lu, 2022), we use work autonomy to reflect the perceived outcome of the work arrangements and policies. Operationally, the latter is a more accurate measure of policy outcomes because flexible working policies may not be well implemented in the practices or vary across groups with different socio-demographic characteristics.

*Occupational class* is measured using the three-category model of the occupational class presented in one version of the National Statistics Socio-economic Classification (NS-SEC). The NS-SEC is one of the most widely used measurements of social class in the UK (Rose & Pevalin, 2003), and was developed from a widely used and reliable measurement of social class, known as the Goldthorpe Schema (Erikson & Goldthorpe, 2010; Savage et al., 2013). In the UKHLS, there is a version of the NS-SEC which presents a three-category model of occupational class: (High) Professional and Managerial class, (Middle) Intermediate class, and (Low) Routine and manual class.

#### Covariates

This study uses the fixed-effects regression models to control all time-constant confounders, even those that are unobserved. Hence, the potential unobserved confounders were restricted to only those that vary over time. The potential observed confounders considered in this study are those that previous research has found to significantly influence the mental health or work-related stress of employees (Chandola et al., 2019; Chandola & Zhang, 2018; Chung & van der Horst, 2020; Li & Wang, 2022; Wheatley, 2017). The final selected demographic confounders include age, the presence of children, long-standing illnesses, the presence and the number of children in the household, marital status, logged household monthly income, job satisfaction and job-related anxiety. Details about these variables are shown in Table 1.

### Analytic Approach

This study uses the fixed-effects regression model, as it is better at identifying causal relations than cross-sectional analysis (Allison, 2009). The results of the Hausman test also suggest using fixed-effects regression instead of random effects. Fixed-effects regression can mitigate any unobserved differences between individuals by measuring only ‘within-individual’ variation (Li & Wang, 2022), such as how changes in work autonomy are linked to changes in employees’ mental well-being. Therefore, this study reaches a more accurate estimate of the relationship between work autonomy and employees’ mental health by eliminating the confounding effects arising from time-constant variables. We first analysed the direct impacts of work autonomy on mental health, and then added the interaction terms between work autonomy and gender, as well as work autonomy and class. The equation for individual fixed effects is as follows:

$${Mental\;well-being}_{it}=\alpha_t+\beta_1{Work\;autonomy}_{it}+\beta_2{Covariates}_{it}+T_t+\mu_i+\varepsilon_{it}$$

where ${Mental\;well-being}_{it}$ refers to the dependent variable for a given individual i at time t where t = 1, 2, …, T; α_t_ refers to the intercept that may vary across time, and ${\beta }_{1}$ is the vector of the coefficients of the independent variable (work autonomy) for individual i at time t. β_2_ is the coefficient for the covariates, T_t_ refers to the effect of time, μ_i_ refers to the time constant error term which will be excluded during the estimation, and ε_it_ refers to the time-varying error term. Then, we also examine whether the presence of children can moderate the impacts of work autonomy on employees’ mental health.

## Results

**Fig. 1:**
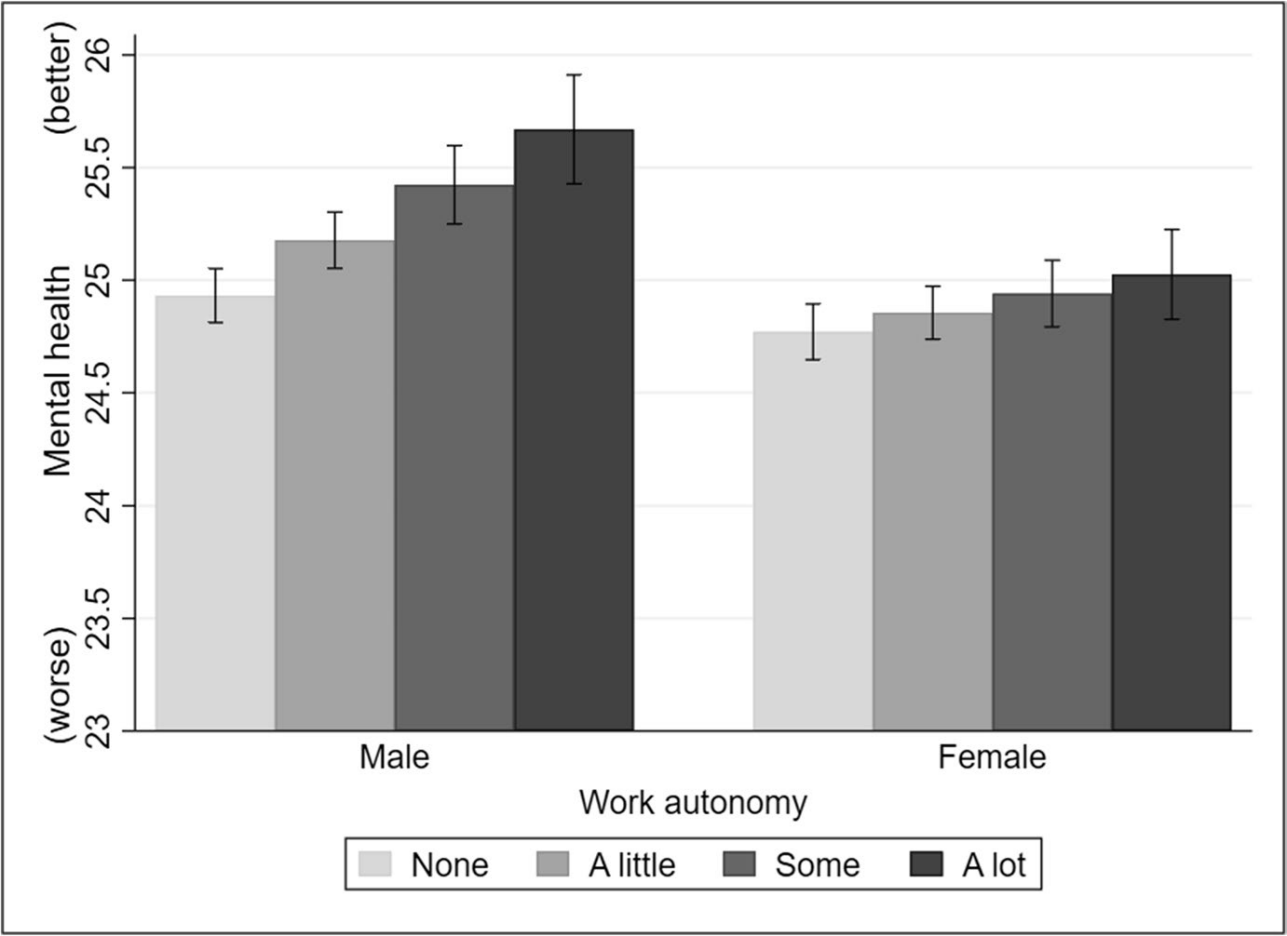
The moderation effects of gender. Note: adjusted predictions of work autonomy and gender with a 95% confidence interval

Table 2 reports the results of several fixed effects models to examine the effects of work autonomy on employees’ mental health and the moderating roles of gender and class. Model 1 identifies the impact of work autonomy on employees’ mental health status. It finds a significantly positive association between work autonomy and mental health (coefficient = 0.15, SE = 0.03, *p* < 0.001), confirming hypothesis 1 that employees with more work autonomy tend to have better mental health status. Model 2 includes an interaction term between work autonomy and gender, which is statistically significant (coefficient = –0.16, SE = 0.05, *p* < 0.01), confirming that gender has significant moderation effects in the association between work autonomy and mental health. To better understand the interaction effects between work autonomy and gender on mental health, we plotted the marginal effects in Fig. 1. As shown in Fig. 1, the positive associations between work autonomy and mental health are more pronounced among males than females. Females’ mental health status can only slightly benefit from higher levels of work autonomy. In sum, these findings are consistent with hypothesis 2, which predicts that the mental benefits of work autonomy on mental health are more pronounced among male employees.

**Table 2:** Fixed effects models examining the effects of work autonomy on employees’ mental health

	Model 1	Model 2	Model 3
Work autonomy	0.15***	0.25***	0.27***
	(0.03)	(0.04)	(0.04)
Occupational class (Ref. = High)			
Middle	0.08	0.08	0.65**
	(0.07)	(0.07)	(0.21)
Low	0.08	0.08	0.58**
	(0.08)	(0.08)	(0.19)
Work autonomy × Sex (Ref. = Male)			
Work autonomy × Female		–0.16**	
		(0.05)	
Work autonomy × Occupational class (Ref. = High)			
Work autonomy × Middle			–0.19**
			(0.06)
Work autonomy × Low			–0.16**
			(0.06)
Constant	25.75***	25.73***	25.38***
	(0.70)	(0.70)	(0.71)
R-squared	0.19	0.19	0.19
Observations	93,903	93,903	93,903

Standard errors are in parentheses. *** *p* < 0.001, ** *p* < 0.01, * *p* < 0.05. All models control for age, logged household monthly income, marital status, the presence of long-standing illness, the presence and the number of children in the household, job satisfaction and anxiety, and wave dummies. See Table 4 in the appendix for more details about the coefficients of the covariates

However, as mentioned in “Occupational Class Disparities” section, the potential moderation effects of occupational class might conceal the real size effect of work autonomy on employees’ mental health between gender. Thus, we also investigated the moderating role of occupational class. Model 3 in Table 2 includes interaction terms between work autonomy and occupational classes, which are statistically significant (work autonomy and middle classes: coefficient = –0.19, SE = 0.06, *p* < 0.01); (work autonomy and low classes: coefficient = –0.16, SE = 0.06, *p* < 0.01). These findings suggest that occupational class significantly moderates the association between work autonomy and mental health. To test the potential intersection between gender and class, we also investigated the three-way interaction between work autonomy, gender and occupational class. The investigation results of the three-way interaction do not support the potential intersection of gender and class (see Table 5 in the appendix). Furthermore, we re-examined the moderation effects of occupational class with gendered samples to capture more nuanced insights into understanding the gender differences in the interaction between work autonomy and occupational class.

**Fig. 2:**
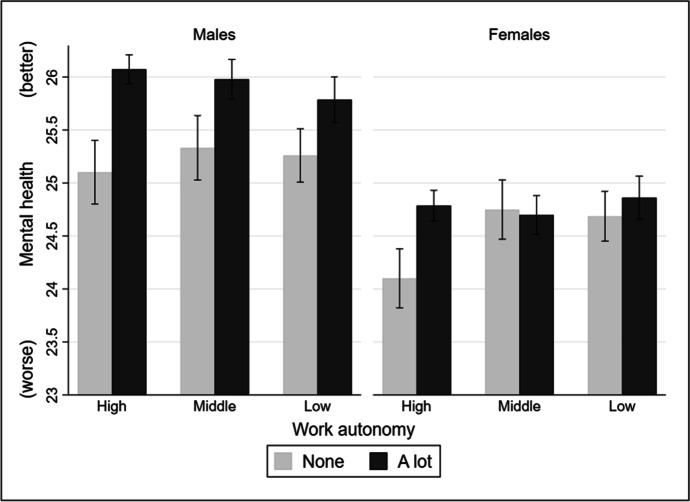
The moderation effects of occupational class (by gender). Note: adjusted predictions of work autonomy and gender with a 95% confidence interval

Table 3 reports the results of several fixed effects models to examine the moderating role of the occupational class by gender. As for males, Model 1 reports insignificant interaction terms between work autonomy and occupational classes. By contrast, as for females, Model 2 reports significant interaction terms between work autonomy and middle classes (coefficient = –0.25, SE = 0.09, *p* < 0.01), and between work autonomy and low classes (coefficient = –0.17, SE = 0.08, *p* < 0.05). Thus, the results in Table 3 indicate that the moderation effects of the occupational class only exist among female employees but not among male employees. To better understand the interaction effects between work autonomy and occupational class by gender, we plotted the marginal effects in Fig. 2. As shown in Fig. 2, on the left side, male employees’ mental health can generally benefit from higher levels of work autonomy with no significant differences between occupational classes. By contrast, on the right side of Fig. 2, the mental benefits of work autonomy are more pronounced among females in the high classes. In addition, females generally have worse mental health status than males. In sum, these findings are partially consistent with hypotheses 3 and 4 but are inconsistent with hypothesis 5. Employees in the higher occupational classes gain more mental-health benefits from work autonomy than their counterparts, while this pattern is only significant among female employees.

**Table 3:** Fixed effects models examining the effects of work autonomy on employees’ mental health (gendered sample)

	Model 1	Model 2
	Men	Women
Work autonomy	0.32***	0.23***
	(0.06)	(0.06)
Occupational class (Ref. = High)		
Middle	0.34	0.89**
	(0.30)	(0.28)
Low	0.31	0.76**
	(0.28)	(0.27)
Work autonomy × Occupational class (Ref. = High)		
Work autonomy × Middle	–0.11	–0.25**
	(0.09)	(0.09)
Work autonomy × Low	–0.15	–0.17*
	(0.09)	(0.08)
Constant	27.05***	24.21***
	(1.05)	(0.96)
R-squared	0.20	0.18
Observations	42,316	51,586

Standard errors are in parentheses. *** *p* < 0.001, ** *p* < 0.01, * *p* < 0.05. All models control for age, logged household monthly income, marital status, the presence of long-standing illness, the presence and the number of children in the household, job satisfaction and anxiety, and wave dummies

## Robustness Check (see Appendix)

A series of tests were conducted to examine the robustness of our results. Firstly, the Hausman test was adopted to see whether we should adopt random-effects regression models instead of fixed-effects regression models. The Hausman test supported the fixed effects regression models (*p* < 0.001). Secondly, additional analyses were conducted to check whether there are problems with treating work autonomy as an integrated continuous variable. Work autonomy can be broadly divided into two categories, namely ‘job control’ and ‘schedule control’ (Wheatley, 2017). Integrated work autonomy was replaced with ‘schedule control’ and ‘job control’ individually, and then the researchers repeated the analyses in the study. The score of employees’ answers to questions about their degree of control over their work hours and pace of work was summed up to calculate a score for employees’ schedule control. Meanwhile, the score of employees’ answers to questions about their degree of control over work tasks, as well as the order and manner in which they are completed, was summed up to calculate a score indicating employees’ job control. The results of the robustness check show that our conclusions remain consistent (see Table 4 in the appendix).

## Discussion and Conclusions

Using nationally representative panel data over a long period (2010–2021) in the UK, this study explores the mental health consequences of work autonomy and its gendered and class-differentiated patterns. The study provides empirical support for the arguments of the Job-Demand Control (JDC) model and the theory of ‘work-family conflict’, emphasising the vital role of work autonomy in employees’ mental well-being and quality of life. In addition, the study builds upon previous studies on work autonomy by considering the moderating effects of employees’ gender and occupational class. It finds that the benefits of work autonomy on employees’ mental health are more pronounced among male employees and female employees in the higher occupational classes. The major theoretical contribution of the article is to bridge hitherto separated strands of literature on work autonomy, gender division of household labour and occupational classes, which offer their respective yet incomplete explanations of women’s low level of mental well-being.

Firstly, we found that work autonomy is indeed a vital indicator of the quality of life, suggesting the theoretical predictions of a linear relationship between work autonomy and workers’ mental health from the JDC model (Karasek, 1979). However, the mental health benefits of work autonomy vary across genders, particularly less pronounced among female employees. This finding partially suggests the arguments from previous studies on the potential adverse effects of work autonomy on women’s mental health, including more multitasking and worse time quality (Chung, 2022; Cornwell, 2013; Craig & Brown, 2017). Specifically, female employees are more likely to use work autonomy to fulfil their family demands and work more due to their disadvantaged positions in both the household and labour market (Clawson, 2014; England et al., 2020). Even though work autonomy can generally promote mental health, its adverse effects on women’s life quality (i.e., role blurring and time-squeeze) (Chung, 2018, 2022) can partially offset the mental benefits. Such gendered disparities in the use and consequence of work autonomy are caused by the remained gender norms and family devotion schema (Chung, 2022; Chung & van der Horst, 2020). Therefore, the study’s findings emphasise that the gender inequalities in the division of labour and the use of work autonomy are pressing matters that must be addressed. Traditional gender norms remain in the labour market and households, partially preventing women from the mental benefits of work autonomy. Policymakers should pay attention to women’s dilemmas and disadvantaged positions in the use of work autonomy and promote more public/occupational benefits (i.e., better accessible and cheap good quality childcare policies) for improving women’s quality of life.

Secondly, the study demonstrates that the mental health benefits of work autonomy can be moderated by occupational class, while the moderating role of the occupational class is only significant among female employees. On the one hand, these findings are consistent with the previous studies on the gendered and class-differentiated division of labour. Though female employees from different occupational groups suffer the ‘flexibility paradox’ in different ways (i.e., more paid work among the higher groups and more unpaid work among the lower groups) (Chung, 2022), the higher occupational groups have advantages in the use of work autonomy than the lower occupational groups. Specifically, previous studies indicate that women working in lower occupational classes have weaknesses in maintaining a work-life balance, as the traditional cultural expectation that women should take on domestic roles is in direct conflict with the ideal worker norms that workers should prioritise their work commitments (Cha & Weeden, 2014). In addition, female employees in higher occupational groups might have more bargaining resources in the division of housework (Kan & He, 2018) and be able to pay for the childcare and housework services, thereby having more options and abilities to juggle work and life when using work autonomy. Thus, work autonomy might not be able to improve the mental health of female employees in the lower occupational groups due to the fact that they are suffering double jeopardy (Wang & Li, 2022). They not only have less work autonomy than higher occupational groups but also suffer more potential adverse effects of work autonomy. On the other hand, these findings are inconsistent with previous studies’ predictions that male employees in the lower occupational classes are less vulnerable to lower work autonomy than their counterparts (Hoven et al., 2015; Kawakami et al., 2004). This might be because male employees in the lower occupational classes have worse work conditions (i.e., less bargaining power and job security) but share heavy financial responsibilities. Thus, men in the lower occupational classes also need work autonomy to alleviate the mental strain brought about by their work and family demands.

This study has some limitations. Firstly, though we use nationally representative samples to ensure external validity in our setting, the findings are limited to the UK and individual levels. However, a growing body of research highlights the linked lives between different family members within households (e.g., couples) (Kim et al., 2019; Wunder & Heineck, 2013) and the different implications of work autonomy across countries (Chung, 2022; Kurowska, 2020; Lott, 2015). Thus, future research could examine the cross-over effects of work autonomy on mental health and its gendered and class-differentiated patterns across different family members and countries. Secondly, a small proportion of our samples cover the Covid-19 period, while we do not capture the mental effects of Coovid-19. However, we control the wave dummies in the analyses, which can control the potential decrease in mental well-being during the pandemic.

These weaknesses should not, however, overshadow the study’s main contributions to the understanding of the consequences of work autonomy on mental well-being across socio-demographic groups. Overall, we found that work autonomy is primarily beneficial to male employees in all occupational classes and female employees in higher occupational groups in terms of mental health. Females in the lower occupational groups are less likely to benefit from work autonomy due to their disadvantaged positions in both the labour market and the household. Therefore, current public policies should be aware of the existence of the moderating role of occupational class in the impacts of work autonomy on female employees’ mental health and the importance of promoting work autonomy for enhancing occupational well-being. The findings in this study are particularly relevant and important in the context of employees’ increasing demands for flexible work and rapid changes in the labour market. The applicability and universality of the conclusions to regions outside the UK depend on regional and cultural gender ideology and specific labour market policies. For countries that have long-period systematically implemented work-family intervention policies and have cultural backgrounds similar to the UK (i.e., Australia, Canada, etc.), the findings of this paper contribute to the guidance of the policy implementation from gender and social class perspectives.

## Data Availability

Data is available from an open-access public depository (accessible at https://www.understandingsociety.ac.uk). The authors would be happy to share the Stata dofile for the data cleaning and analysis of the study. Please email the corresponding author to obtain the materials.

## Appendix

Table 4

**Table 4:** Fixed effects models examining the effects of work autonomy/job control/schedule control on employees’ mental health (with the coefficients of the covariates)

	Work autonomy	Job control	Schedule control
	Model 1	Model 2	Model 3
Work autonomy	0.15***	0.15***	0.10***
	(0.03)	(0.02)	(0.03)
Occupational class (Ref. = High)			
Middle	0.08	0.08	0.07
	(0.07)	(0.07)	(0.07)
Low	0.08	0.08	0.06
	(0.08)	(0.08)	(0.08)
Age	0.09	0.08	0.09
	(0.05)	(0.05)	(0.05)
Age squared	0.00***	0.00***	0.00***
	(0.00)	(0.00)	(0.00)
Marital status (Ref. = Never married)			
Married	0.27**	0.27**	0.27**
	(0.09)	(0.09)	(0.09)
Divorced/separated/widowed	–0.05	–0.06	–0.06
	(0.13)	(0.13)	(0.13)
The presence of dependent children (Ref. = No)			
Yes	–0.13	–0.13	–0.14
	(0.08)	(0.08)	(0.08)
Long-standing illness (Ref. = Yes)			
No	0.57***	0.57***	0.57***
	(0.05)	(0.05)	(0.05)
Number of children in the household	0.00	0.01	0.00
	(0.04)	(0.04)	(0.04)
Log household income (monthly)	0.13**	0.13**	0.14**
	(0.05)	(0.05)	(0.05)
Job satisfaction	0.36***	0.36***	0.37***
	(0.01)	(0.01)	(0.01)
Job anxiety	–2.23***	–2.23***	–2.23***
	(0.03)	(0.03)	(0.03)
Constant	25.17***	25.16***	25.32***
	(0.70)	(0.70)	(0.70)
R-squared	0.19	0.19	0.19
Observations	93,903	93,903	93,903

Note: Standard errors in parentheses. *** *p* < 0.001, ** *p* < 0.01, * *p* < 0.05

Table 5

**Table 5:** The three-way interaction between work autonomy, occupational class, and sex

	Model 1
Work autonomy	0.33***
	(0.07)
Work autonomy × Sex (Ref. = Male)	
Work autonomy × Female	–0.11
	(0.09)
Occupational class (Ref. = High)	
Middle	0.34
	(0.32)
Low	0.37
	(0.30)
Work autonomy × Occupational class (Ref. = High)	
Work autonomy × Middle	–0.10
	(0.10)
Work autonomy × Low	–0.16
	(0.09)
Sex × Occupational class (Ref. = Male × High)	
Female × Middle	0.53
	(0.42)
Female × Low	0.35
	(0.39)
Work autonomy × Sex × Occupational class (Ref. = Male × High)	
Work autonomy × Female × Middle	–0.13
	(0.14)
Work autonomy × Female × Low	0.04
	(0.13)
Constant	25.37***
	(0.71)
R-squared	0.19
Observations	93,903

Standard errors in parentheses. *** *p* < 0.001, ** *p* < 0.01, * *p* < 0.05
